# Association between antimicrobial resistance among Enterobacteriaceae and burden of environmental bacteria in hospital acquired infections: analysis of clinical studies and national reports

**DOI:** 10.1016/j.heliyon.2019.e02054

**Published:** 2019-07-22

**Authors:** Thor-Henrik Henriksen, Workeabeba Abebe, Wondwossen Amogne, Yitagesu Getachew, Harald Weedon-Fekjær, Jörn Klein, Yimtubezinash Woldeamanuel

**Affiliations:** a Department of Internal Medicine, School of Medicine, Addis Ababa University, Ethiopia; b Department of Internal Medicine, Yekatit 12 Hospital Medical College, Ethiopia; c Department of Microbiology, Vestfold Hospital Trust, 3103, Tönsberg, Norway; d Department of Pediatrics and Child Health, School of Medicine, Addis Ababa University, Ethiopia; e Oslo Center for Biostatistics and Epidemiology, Research Support Services, Oslo University Hospital, Oslo, Norway; f Faculty of Health and Social Sciences, University of South-Eastern Norway, Kongsberg, Norway; g Department of Microbiology, Immunology and Parasitology, School of Medicine, College of Health Sciences, Addis Ababa University, Addis Ababa, Ethiopia

**Keywords:** Infectious disease, Antimicrobial resistance, Antibiotic resistance, Global health, Africa, Europe, Extended spectrum beta-lactamase, Gram-negative bacteria, Acinetobacter spp., Enterobacteriaceae, Antimicrobial resistance vulnerability

## Abstract

**Background:**

WHO has named three groups of gram-negative bacteria “our critical antimicrobial resistance-related problems globally”. It is thus a priority to unveil any important covariation of variables behind this three-headed epidemic, which has gained alarming proportions in Low Income Countries, and spreads rapidly. Environmental bacteria including Acinetobacter spp. are common nosocomial pathogens in institutions that have high rates of antimicrobial resistance among other groups of gram-negative bacteria.

**Methods:**

Based on two different data sources, we calculated the correlation coefficient (Pearson's *r*) between pathogenic burden of Acinetobacter spp. and antimicrobial resistance among Enterobacteriaceae in European and African nosocomial cohorts.

**Clinical reports:**

Database search for studies on nosocomial sepsis in Europe and Africa was followed by a PRISMA-guided selection process.

**National reports:**

Data from Point prevalence survey of healthcare-associated infections published by European Centre for Disease Prevention and Control were used to study the correlation between prevalence of Acinetobacter spp. and antimicrobial resistance among *K. pneumoniae* in blood culture isolates.

**Findings:**

The two approaches both revealed a strong association between prevalence of Acinetobacter spp. and rates of resistance against 3. generation cephalosporins among Enterobacteriaceae. In the study of clinical reports (13 selected studies included), *r* was 0.96 (0.80–0.99) when calculated by proportions on log scale. Based on national reports, *r* was 0.80 (0.56–0.92) for the correlation between resistance rates of *K. pneumoniae* and proportion of Acinetobacter spp.

**Interpretation:**

The critical antimicrobial resistance-related epidemics that concern enteric and environmental gram-negative bacteria are not independent epidemics; they have a common promoting factor, or they are mutually supportive. Further, accumulation of antimicrobial resistance in nosocomial settings depends on the therapeutic environment. Burden of Acinetobacter spp. as defined here is a candidate measure for this dependence.

## 1 Introduction

In Africa we have experienced a landslide of antimicrobial resistance (AMR) among gram-negative bacteria (GNB) and the clinical consequences of this misfortune, while three groups of gram-negative bacteria have been named critical antimicrobial resistance-related threats globally by WHO [1, 2, 3, 4]. The related AMR situation is still astonishingly muted and manageable in affluent countries [5, 6]. Improved insight into global health-related aspects is now necessary to understand the circumstances that made not less than three different groups of GNB the uppermost AMR-related threats worldwide [4].

Antimicrobial resistance is accumulated when bacteria are exposed to antibiotics. However, while variations are noted, the association between consumption of antimicrobials and antimicrobial resistance is generally weak [7]. Antimicrobial resistance also depends on factors that have so far not been accounted for [8, 9]. To understand their importance, we obviously need to make them countable, but we lack the needed measure.

Through our work in Europe and Africa, we have noticed that environmental bacteria, in particular Acinetobacter spp., are common pathogens in nosocomial sepsis cohorts that also have exceptionally high rates of resistant Enterobacteriaceae [2]. This impression was strengthened through unstructured review of reports from others [10, 11, 12]. Any association between disease burden of Acinetobacter spp. and rates of resistance among Enterobacteriaceae would be of major epidemiological importance:

- It would mean that the critical epidemics of AMR related to Enterobacteriaceae and environmental bacteria are not independent epidemics; they would either be mutually supportive, or have a promoting factor in common.
- Association between AMR among enteric bacteria and a variable that belongs to the environment (Acinetobacter spp.) would link magnitude of AMR to one measurable element within the environment. This would make Acinetobacter spp. a surrogate marker and a candidate measure for impact of the therapeutic environment on accumulation of AMR.

We have therefore studied the association between burden of Acinetobacter spp. as nosocomial pathogens and antimicrobial resistance among Enterobacteriaceae in clinical reports from Europe and Africa. To assess the quality of evidence in case of a positive correlation, we also analysed the association based on entirely different data, i.e. from national European records that were published by the European Centre for Disease Prevention and Control (ECDC).

The first part thus concerned clinical studies from Europe and Africa. During the preparatory phase, we became aware of a number of challenges. While recommendations for increased stringency in research were observed [13, 14, 15], clinical discrepancies of major epidemiological importance were commonly not accounted for. Levels of antimicrobial resistance differ greatly, not only between cohorts with community-acquired (CAI) and hospital acquired infections (HAI), but also between invasive and non-invasive infections [3, 6]. Correct handling of bacteriological and susceptibility-related results would thus be separate specimen-dependent tables for HAI and CAI cohorts. Because sufficiently detailed tables were commonly not offered, we adopted a review procedure that included in depth review of text and tables. Following this, a number of studies were found to have result entries that were either incorrect (mixture of patients' cohorts), or missing. To our knowledge, standard procedures that are sufficiently detailed about management of such cases do not exist [13, 14, 15]. This was thus handled as described below.

African reports were indispensable in the present study, but previous review studies have all failed to include reports from Africa on invasive nosocomial infections [16]. There are reviews on blood stream infections that include African studies, but they do not contain cases of hospital-acquired infection [17]. Then there are reviews on hospital-acquired infections that include African studies, but they do not contain African cases of invasive infections [18, 19]. Through thorough examination as described above, we identified relevant African studies that fulfilled the eligibility criteria.

For the study of national reports, sources of aggregated data were sought for. While the European Centre for Disease Prevention and Control (ECDC) has published reports about AMR rates for relevant pathogens in most European states for a number of years, proportion of Acinetobacter spp. as nosocomial pathogens could only be found in one point prevalence study (PPS) presenting data from 2012 [5,20,21]. The second ECDC PPS was organized in 2016–2017, but data published so far do not contain prevalence of nosocomial pathogens.

Relating antimicrobial resistance among Enterobacteriaceae to the prevalence of a different group of bacteria has not been done before. There are thus no previous reports for comparison. Instead, related methodological, biological and epidemiological aspects were discussed.

## 2 Methods

Throughout the study, reported resistance were rates of antimicrobial resistance towards third generation cephalosporins (3GC). The highest number reported for resistance to any one of ceftazidime, ceftriaxone, cefotaxime, or report of ESBL, was recorded.

### 2.1 Review of HAI studies/reports from European and African institutions

PRISMA flow diagram was followed [22]. The applied database was Ovid MEDLINE, and the search was performed by qualified librarians. The search strategy itself included clinical (sepsis, septic?emi*, bacter?emi*, bloodstream, blood stream infection), cohort-related (hospital-acquired, HAI, nosocomial infection), geographical [continent Africa plus all African countries by name; continent Europe plus three South-European (Greece, Italy, Spain) and four North- European countries (Norway, Sweden, Finland, Denmark) by name], as well as time-related aspects (2010-current). The mentioned countries were selected to ensure inclusion of studies from areas with high, middle and low prevalence of antimicrobial resistance.

Review of all abstracts and full texts as well as the following decisions were made by two or more authors. HAI studies with reports on blood culture isolates, or general sepsis studies that also contained these informations as a subgroup were selected. These were reviewed in full text to identify those from which the following information could be provided either directly, or derived indirectly:

• number of Acinetobacter spp. isolates,
• Total number of GNB isolates, and
• rate of 3GC resistant Enterobacteriaceae isolates.

Minimum accepted study size was defined by GNB = 30 cases. Multicenter studies were included, even those involving centers outside Europe and Africa provided that institutions from either of these two continents were also included.

Articles that met these criteria were subjected to final quality check of data. This was done to confirm the following:

• cases belonged to target cohort (HAI/HCAI cases),
• correct specimen for all cases (blood cultures),
• number of missing or doubtful entries for all target parameters.

Reports were accepted when missing or doubtful entries for any parameter concerned were ≤15%. Doubtful entries would be doubtful or possible CAI cases, and those where kind of specimen could not be ascertained. Care was made to ensure correct number for Acinetobacter spp. Studies reporting of non-fermenting bacteria without further identification were thus only accepted when this group of bacteria was ≤15% of those already identified as Acinetobacter spp. There was one exception, however, where the rate of non-Pseudomonas non-fermenters was exceptionally low, i.e. 2% [33]. In that case, proportion of Acinetobacter spp. was given the value 1%. This study was included as explained further down.

Authors were asked for additional information when missing or doubtful entries concerned >15 % - 50% of entries for any given variable. Inclusion would then depend on the author's answer. Reports were excluded when missing or doubtful entries concerned the majority of entries for any variable.

While community acquired infections (CAI) were thus weeded out, cases defined by the author as health care associated infection (HCAI) were counted as HAI cases. Lack of susceptibility test results for Enterobacter spp., was expected and accepted [23, 24].

The fraction of Acinetobacter spp. by total number of GNB isolates was named Proportion of Acinetobacter spp. Correlation between Proportion of Acinetobacter spp. and rate of 3GC resistant Enterobacteriaceae was then calculated, both as simple correlation of proportions and on log scale.

### 2.2 National reports

This part was based on data from European Antimicrobial Resistance Surveillance (EARS)/European Centre for Disease Prevention and Control (ECDC) [5, 20, 21]. We recorded rates of Acinetobacter spp. as they were given in “Point prevalence survey (PPS) of healthcare-associated infections and antimicrobial use in European acute care hospitals 2011–2012” (isolates of Acinetobacter spp. by total number of HAI isolates), and rates of resistant *K. pneumonia* in national blood culture records from countries that reported to EARS in 2012 [36,38].

Correlation coefficient (Pearson's *r*) and *r*^*2*^ for the association between prevalence of Acinetobacter spp. and rate of 3GC resistant *K. pneumonia* were then calculated.

## 3 Results

### 3.1 Review of HAI studies

**Fig. 1:**
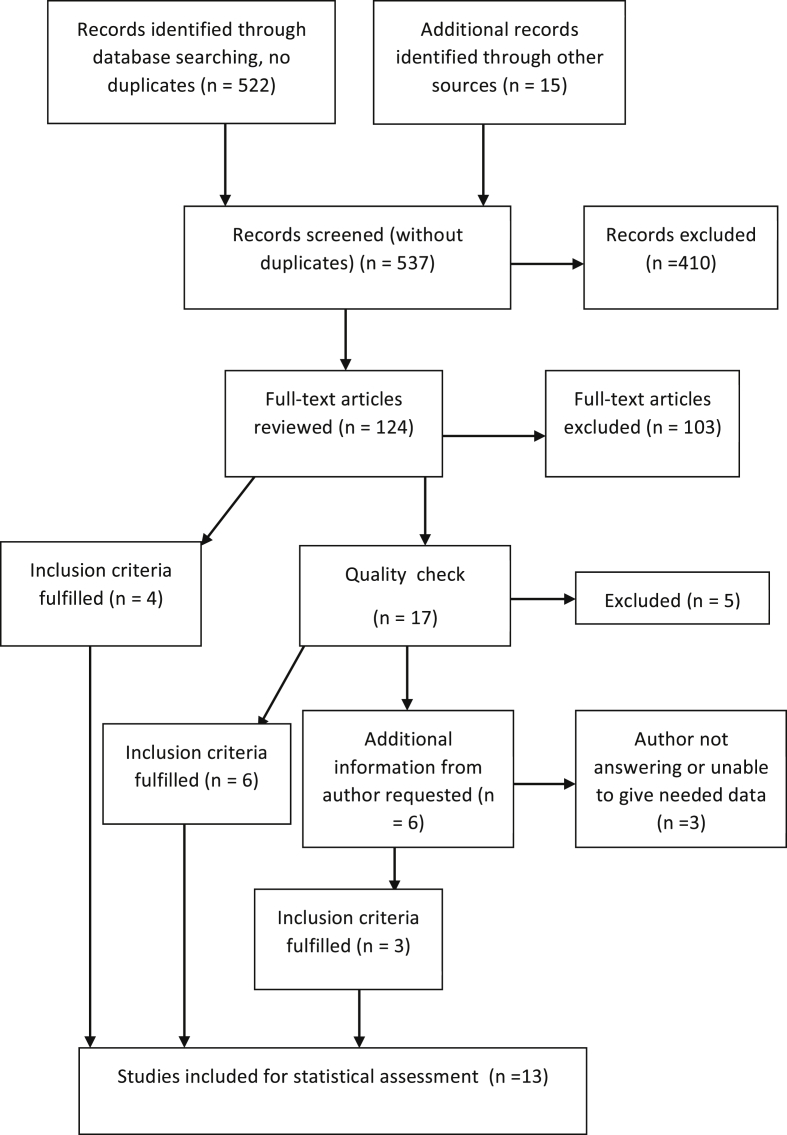
Selection process.

**Fig. 2:**
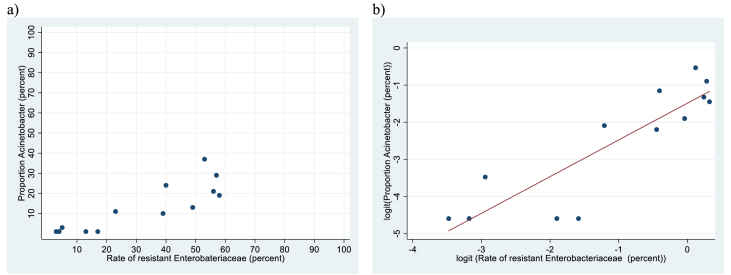
Correlation between Proportion of Acinetobacter spp. (see text), and resistance towards third generation Cephalosporins among Enterobacteriaceae; Left (a) absolute proportions, right (b) proportions on log scale with fitted regression line. (Based on reports from European and African institutions).

Outcome of the search and selection process is given in Fig. 1. The database search yielded 522 articles, to which 15 more were obtained via citation chasing. Following the abstract review, 124 selected reports were assessed in full text for eligibility. Of these, 103 were excluded because they did not meet the eligibility criteria. In four of the studies, all needed variables were easily retrieved from the tables, and these studies were included immediately. Crucial characteristics, comments and result are given in Table 1 for these four and the remaining 17 studies that were subjected to the final quality check [3, 25, 26, 27, 28, 29, 30, 31, 32, 33, 34, 35, 36, 37, 38, 39, 40, 41, 42, 43, 44. Six of these 17 studies were included following cross-check of text and tables. One study was excluded because cohort affiliation (HAI or CAI) for majority of susceptibility test results could not be accounted for [3]; two because susceptibility results for Enterobacteriaceae were not given [30, 31]; one study because susceptibility test had not been performed for the majority of isolates [39]; one because it was a sub-study, and the main study was already included [32]. In the remaining six cases, authors were asked to provide for additional information about exact number of resistant isolates of a given species [28, 33, 36, 37], or number of *Acinetobacter* isolates [29, 43]. Three of the authors gave the needed information which lead to the inclusion of their studies. Two refrained from answering while one author told that he was unable to give the needed data. There were thus 13 studies for calculation of the correlation coefficient (Table 2 and Fig. 2).

**Table 1:** Crucial data and outcome of quality check.

Ref	First Author	Country	Institution	Patients' cohorts	Sampling year	Pending issue, and outcome of quality check.	Metho-dology	GNB (no)	Entero-bacteriaceae (no)	Resistance rate Entero-bateriaceae	Proportion of Acinetobacter (%)	Quality
[3]	Maina	Kenya	Aga Khan Gen.Hospital Nairobi	NICU, Paediatric, Medical, Surgical	2010-14	Susceptibility test results given for HAI and CAI combined, 70% being CAI. **Not inclued**	CLSI	189	152	NG	11	-
[25]	Morkel	South Africa	Tygerberg Childrens Hospital Cape Town	Children including neonates	2008	15% of specimen harvested < 48 h after admission or birth. **Included**	CLSI	38	23	53	37	+
[26]	Dramowski	South Africa	Tygerberg Childrens Hospital Cape Town	Neonates	2009-13	Susceptibility of Enterobacteriaceae concerns *Klebsiellae pneumoniae* and *E. coli* only (=88,5% of all Enterobacteriaceae) **Included**	CLSI	519	426	49	13	+
[27]	McKay	South Africa	Groote Schuur Hospital, Cape Town	Adult patients	2011-12	**Included**	CLSI	366	214	40	24	+
[28]	Zorgani	Libya	Burn and Plastic Surgery Centre	Burn cases	2000 - 2007	Missing susceptibility test results. (Author’s answer). **Not inclued**	NG	170	57	NG	6	-
[29]	Saied	Egypt	Multicentre	3 univ hosp	2006-7	No. of *Acinetobacter spp*. uncertain: 29 isolates identified, in addition 33 "Nonfermenter spp", i.e. proportion of Acinetobacter 7,5 - 16,1. **Not inclued**	CLSI	386	267	66	NG	-
[30]	See	Egypt	Multicentre	46 egyptian ICUs, all ages(incl 8 NICUs)	2011-12	Only 88/362 isolates (24%) sent for susceptibility test. **Not inclued**	CLSI	51	29	NG	13	-
[31]	Talaat	Egypt	Incl. 91 ICUs, 28 Hospitals in Egypt.	NICU, Paediatric, Medical, Surgical, Neurological	2012-14	Microbes and susceptibility results given for all types of samples/specimen in one. **Not inclued**	CLSI	NG	NG	NG	NG	-
[42]	Lachhab	Morocco	Mohammed V Military Teaching Hospital	ICU	2012 - 13	**Included**	NG	45	25	57	29	+
[38]	Mitt	Estonia	PICU, Tartu University Hospital	PICU	2004-8	**Included**	CLSI	47	39	23	11	+
[43]	Horcajada	Spain	8 Spanish ICUs	Unselected sepsis patients	2010-11	Author’s clarification about number of Acintobacter isolates. **Included**	CLSI	371	339	13	1	+
[33]	Bartoletti	Italy	Orsola-Malpighi Hospital Bologna, Italy	8874 patients with cirrhosis	2008-12	Missing test results provided by author. **Included**	CLSI / EUCAST	104	77	39	10	+
[44]	Softic	Bosnia-Herzegovina	University Clinical Centre Tuzla	NICU	2012 - 13	**Included**	CLSI	32	19	58	19	+
[40]	Reunes	Belgium	Ghent University Hospital	Geriatric patients	1992-2007	**Included**	NCCLS	76	61	5	3	+
[34]	De Bus	Belgium	Ghent University Hospital	Unselected HAI cohort	2009 - 11	Susceptibility results given as mean for all GNB, but 86% of GNB are Enterobacteriaceae. **Included**	NG	565	487	17	1	+
[41]	Tabah	Europe	162 intensive care units (ICUs) in 24 countries.	Unselected HAI cohorts	2009	**Included**	NG	759	342	56	21	+
[39]	Perez Lopez	UK	St. George’s Hospital NHS Trust,	Pediatric/NICU	2001-9	Only 33% of all Enterobacteriaceae tested for suceptibility. **Not inclued**	BSAC	108	78	NG	6	-
[37]	Melzer	UK	Royal London Hospital, Barts Health NHS Trust,	UTI associated bacteremia	2007-8	Report of 6 ESBL positives in subset of 83 isolates - number of tested isolates not given. **Not inclued**	BSAC	71	62	NG	0	-
[32]	Åttman	Finland	Tampere University Hospital	Haematho-logical malignancies	1999-2001 and 2005-07	Study population is part of other included study. **Not inclued**	CLSI / EUCAST	171	115	6	0	-
[35]	Huttunen	Finland	Tampere University Hospital	Unselected HAI cohort	1999-2001 and 2005-07	Acinetobacter-rate not given, but no. of non-Pseudomonas non-Enterobacteriaceae GNB was 11, i.e. 2,0% of GNB. Proportion of Acinetobacter defined by us as 1%. **Included**	CLSI / EUCAST	548	450	3	1	+
[36]	Mehl	Norway	Nord-Trøndelag Hospital Trust	Unselected sepsis cases	2002 - 13	Missing susceptibility results supplied on demand. **Included**	EUCAST	586	483	4	1	+

Ref: Reference; no: number; NICU: Newborn Intensive Care Unit; HAI: Hospital acquired infection; CAI: Community acquired infection; CLSI: Clinical & Laboratory Standards Institute; EUCAST: European Committee on Antimicrobial Susceptibility Testing; BSAC: British Society for Antimicrobial Chemotherapy; NCCLS: National Committee for Clinical Laboratory Standards; NG: not given; ICU: Intensive Care Unit; PICU: Pediatric Intensive Care Unit; GNB: Gram-negative bacteria; UTI: Urinary tract infection.

**Table 2:** Correlation between proportion of Acinetobacter spp. and rates of antimicrobial resistance among Enterobateriaceae, from simple correlation to more statistically sound calculations on log scale accounting for observation uncertainty due to limited number of samples. (Based on reports from European and African institutions).

Measurement	Correlation with 95% confidence interval∗
a) Basic correlation of proportions	0.85 [0. 74, 0.95]
b) Correlation of proportions on log scale	0.88 [0.78, 0.97]
c) Correlation of proportions on log scale; accounting for observation uncertainty∗∗	**0.96 [0.80, 0.99]**

∗) Confidence intervals for (a) and (b) calculated by bootstrap replications.

∗∗) Calculated using Bayesian Gibbssampler assuming multivariate normal distributions and flat priors.

The correlation coefficient was high with all applied methods. On log-log scale, the correlation coefficient was 0.88. In one included study, the proportion of Acinetobacter spp. was <2%, and given the value 1% by us (Fig. 2, Table 2) [35]. The correlation coefficient would have been 0.85 and 0.89, respectively, if the given value for proportion of Acinetobacter spp. in the concerned study had been 2% or 0.1% instead [35]. When calculated by proportions on log scale accounting for observation uncertainty due to limited number of samples, Pearson's *r* was 0.96 (0.80, 0.99).

### 3.2 National reports

The correlation coefficient (*r*) between rates of resistance among *K. pneumoniae* and proportion of Acinetobacter spp. was 0.80 (0.56, 0.92) [*r*^*2*^ = 0.64 (0.31, 0.84)]

## 4 Discussion

The correlation between prevalence of Acinetobacter spp. and rates of antimicrobial resistance among Enterobacteriaceae was very high and had a narrow confidence interval that reached 0.99. Ninety-two per cent of the observed resistance among Enterobacteriaceae could thus be explained by the corresponding burden of Acinetobacter spp. (*r*^*2*^) based on the review of clinical studies. The evidence quality consisted of the strength of this correlation, plus the fact that equal results were reached through two entirely different approaches: clinical studies and national reports [45].

The understanding is that the simultaneous epidemics of AMR among Enterobacteriaceae and non-fermenting bacteria are related. Either, these epidemics have a common promoting factor, or they are mutually supportive.

Further, this is the first report of a link between resistance patterns among enteric bacteria and the disease burden of bacteria that originate from the therapeutic environment itself. Different aspects of hospital operation that affect burden of Acinetobacter spp., are of equal importance for promotion or prevention of AMR build-up.

### 4.1 Antimicrobial resistance among enteric bacteria covariates with level of nosocomial pathogens originating from the environment. Which are the implications?

The association between a family of nosocomial pathogens (Acinetobacter spp.) and levels of antimicrobial resistance among Enterobacteriaceae highlights the complexity of AMR accumulation. The risk for hospital-acquired infections increases when advanced medical and surgical procedures are introduced, and so does the levels of antimicrobial resistance [18, 46]. It was unfortunate, but probably not an accident, that increased rates of antimicrobial resistant GNB coincided with the introduction of advanced medical and surgical procedures in Africa, and Low and Middle Income Countries (LMIC) elsewhere [47]. Not counted as prestigious activities, bacteriological services and hospital hygiene have low priority, while cardiovascular and other advanced surgical services are now being introduced [48, 49]. Symbol equipment like ventilators are introduced as hardly affordable and very unfortunate substitutes for safe and comprehensive intensive care [50]. As highlighted here, hospital management without bacteriological services is no longer an option. It is always unfortunate when the level of advanced and device-dependent medical care is not balanced against the institutions’ ability to record and manage problems that follow advanced medical care [51]. In health care settings where non-fermenters and other contaminants are constantly sought for and immediately removed whenever found, any co-existence between Enterobacteriaceae and non-fermenting bacteria would be occasional and of short duration. In the absence of bacteriological services and strong hospital hygiene, however, contaminants may unnoticed live as endemic parts of the environment [19].

### 4.2 Amplified build-up of antimicrobial resistance: synergy between non-related gram-negative bacterial species after all?

The simultaneous epidemiological success of three different groups of gram-negative bacteria has now forced us to study the significance of differently composed multi-bacterial environments for production of AMR [7, 52, 53]. Enhanced acquirement and propagation of AMR by non-fermenting bacteria, and reception of these elements by Enterobacteriaceae, have all been described [52, 54, 55]. Theoretically, AMR production could thus be amplified when mutually complementary bacterial properties are brought together.

Horizontal gene transfer (HGT) of ESBL between Enterobacteriaceae and Acinetobacter spp. has not been regarded as a matter of importance. Class A beta-lactamases, common to Enterobacteriaceae, are seldom found among Acinetobacter spp [56, 57, 58]. While Acinetobacter spp. thus do not keep Class A beta-lactamases themselves, to some extent they do acquire and propagate plasmids that contain the concerned sequences [52, 59, 60, 61]. The significance of this contribution to build-up of AMR within a multi-bacterial environment has not been studied, but this can be done. It is now possible to follow transfer of AMR from donor to receiving bacteria [62, 63].

Shared plasmids and shared clonal resistance among different species has not been extensively studied [62, 64, 65]. It could nevertheless be widespread, both across species borders and geographical borders. In 2003, 13 different blood culture isolates, representing 4 different species (all Enterobacteriaceae) were harvested among Tanzanian children [66, 67]. The 13 isolates had one common plasmid, which carried resistance towards 5 antibiotics (3GC, gentamicin, chloramphenicol, co-trimoxazole and tetracycline). When looked after ten years later, we found that the majority of 92 pediatric GNB blood culture isolates in Addis Ababa, Ethiopia, expressed equal AMR phenotypes. This resistance pattern was found regardless of species, 32% of isolates being non-fermenting bacteria [2].

At present, the most important AMR-related task is thus to understand how three groups of gram-negative bacteria became our three critical AMR-related problems. We have shown that two of them are linked together, and thus not independent epidemics. While antimicrobial consumption remains the trigger of AMR accumulation, the extent of AMR accumulation depends on the antimicrobial resistance vulnerability of the nosocomial environment. We have offered a candidate measure for such vulnerability.

### 4.3 Needed research

Research related to AMR needs reorientation: thematically to focus on the different elements that amplify AMR production, geographically to reach study populations where AMR build-up is fast, and bacteriologically to secure that WHO's three critical issues are granted top priority.

More detailed, further research is needed to identify the forces behind the rapid and simultaneous epidemiological success of three groups of gram-negative bacteria. As part of this, we need to know if co-existence between Acinetobacter spp. and Enterobacteriaceae affects rates of AMR. Then the following questions need to be answered: Is burden of Acinetobacter spp. a useful measure for the environment's impact on AMR build-up in hospitals? Are antimicrobial stewardship programs at all helpful when rates of AMR and burden of Acinetobacter spp. are both high - or should improved hospital hygiene be addressed to reduce the burden of Acinetobacter spp. first? Is co-existence of gram-negative bacterial species with mutually complementary properties for acquisition and propagation of plasmids optimal for spread of AMR?

It has previously been stated that “large-scale dissemination of multi-resistant pathogens in the hospital environment, the community, and the wider environment is one of the most important emerging public health threats” [54]. The distinction between these three levels is probably as important as their relatedness. We need to understand the process that transforms health care facilities into sources of AMR, where after AMR is spread into the catchment areas and beyond by Enterobacteriaceae. Having reached out into the community, AMR rates are further increased through unstructured and high use of antibiotics [68, 69, 70, 71]. When thresholds for hospital admission are as high as they are unpredictable, room is created for unstructured and unpredictable pre-hospital management and AMR build-up [72]. While working on this project, the main challenge was poor availability of reliable data. In particular, we missed tables of antimicrobial susceptibility results with respect to species, kind of specimen, clinical data, and matching cohort. If appropriate datasets had followed reports as attachments, this problem would have been solved.

### 4.4 Limitations

#### 4.4.1 Clinical studies

Because of the highly detailed review process, we became aware of studies with missing or incorrect data entries for one or more of the variables. We decided to accept studies when missing or incorrect entries were random and to low to be of importance - defined as 15% of all entries for a given variable. We were not aware of any standard procedure for how to handle this, and the method we developed and used had thus not been validated or applied before.

To manage the detailed review as described above in a proper way, a database search procedure was used that would leave out maximum of irrelevant or low quality reports. It is possible that a broader search could have given an even higher number of included studies.

#### 4.4.2 National reports

The ECDC report did not contain breakdowns for causative agents in blood cultures. We therefore applied the reported proportion for *Acinetobacter* spp. for all specimens, while rates of AMR concerned blood culture results only. A more reliable and probably an even stronger correlation would have been the result if proportion for *Acinetobacter* spp. had solely been based on blood culture reports in HAI.

Quality of bacteriological methods including species identification, susceptibility tests and ESBL identification was not assessed.

## Declarations

### Author contribution statement

Thor-Henrik Henriksen: Conceived and designed the study; Participated in the data selection process; Analyzed and interpreted the data; Supervised the process; Wrote the paper.

Workeabeba Abebe, Wondwossen Amogne, Jörn Klein: Participated in the data selection process; Analyzed and interpreted the data; Wrote the paper.

Yitagesu Getachew: Analyzed and interpreted the data; Participated in the data selection process; Wrote the paper.

Harald Weedon-Fekjær: Selected statistical methods; Performed statistical calculations; Analyzed and interpreted the data; Wrote the paper.

Yimtubezinash Woldeamanuel: Analyzed and interpreted the data; Wrote the paper.

### Funding statement

This research did not receive any specific grant from funding agencies in the public, commercial, or not-for-profit sectors.

### Competing interest statement

The authors declare no conflict of interest.

### Additional information

No additional information is available for this paper.
